# A Couples-Based Intervention (Ghya Bharari Ekatra) for the Primary Prevention of Intimate Partner Violence in India: Pilot Feasibility and Acceptability Study

**DOI:** 10.2196/26130

**Published:** 2021-02-01

**Authors:** Ameeta Shivdas Kalokhe, Sandhya Iyer, Keshav Gadhe, Tuman Katendra, Ambika Kolhe, Girish Rahane, Rob Stephenson, Seema Sahay

**Affiliations:** 1 Division of Infectious Diseases Department of Medicine Emory University School of Medicine Atlanta, GA United States; 2 Department of Global Health Emory Rollins School of Public Health Atlanta, GA United States; 3 Department of Social and Behavioral Research National AIDS Research Institute Pune India; 4 Department of Systems, Population and Leadership University of Michigan School of Nursing Ann Arbor, MI United States

**Keywords:** intimate partner violence, prevention, pilot study, gender-based violence, domestic violence, violence, India, intervention, prevalence, mental health, acceptance, safety, feasibility, efficacy

## Abstract

**Background:**

The high global prevalence of intimate partner violence (IPV) and its association with poor physical and mental health underscore the need for effective primary prevention. We previously developed Ghya Bharari Ekatra (GBE), a couples-based primary prevention intervention for IPV among newly married couples residing in slum communities in Pune, India.

**Objective:**

Through this pilot study, we aimed to explore the acceptance, safety, feasibility, and preliminary efficacy of GBE.

**Methods:**

Between January and May 2018, we enrolled and assigned 20 couples to receive GBE plus information on IPV support services and 20 control couples to receive information on IPV support services alone. The GBE intervention was delivered over 6 weekly sessions to groups of 3 to 5 couples by lay peer educators in the communities in which the participants resided. Intervention components addressed relationship quality, resilience, communication and conflict negotiation, self-esteem, sexual communication and sexual health knowledge, and norms around IPV. Outcome evaluation included exit interviews with participants and peers to examine acceptance and feasibility challenges and baseline and 3-month follow-up interviews to examine change in IPV reporting and mental health (by women) and alcohol misuse (by men). The process evaluation examined dose delivered, dose received, fidelity, recruitment, participation rate, and context.

**Results:**

Half (40/83) of the eligible couples approached agreed to participate in the GBE intervention. Retention rates were high (17/20, 85% across all 6 sessions), feedback from exit interviews suggested the content and delivery methods were very well received, and the community was highly supportive of the intervention. The principal feasibility challenge involved recruiting men with the lowest income who were dependent on daily wages. No safety concerns were reported by female participants over the course of the intervention or at the 3-month follow-up. There were no reported physical or sexual IPV events in either group, but there were fewer incidents of psychological abuse in GBE participants (3/17, 18%) versus control participants (4/16, 25%) at 3-month follow-up. There was also significant improvement in the overall mental health of female intervention participants and declines in the control participants (change in mean General Health Questionnaire-12 score: –0.13 in intervention vs 0.13 in controls; *P*=.10).

**Conclusions:**

GBE has high acceptance, feasibility, and preliminary efficacy in preventing IPV and improving mental health among women. Next steps include refining the intervention content based on pilot findings and examining intervention efficacy through a large-scale randomized trial with longer follow-up.

**Trial Registration:**

ClinicalTrials.gov NCT03332134; https://clinicaltrials.gov/ct2/show/NCT03332134. Clinical Trials Registry of India CTRI/2018/01/011596; http://ctri.nic.in/Clinicaltrials/pmaindet2.php?trialid=21443

**International Registered Report Identifier (IRRID):**

RR2-10.2196/11533

## Introduction

Intimate partner violence (IPV), defined by the World Health Organization (WHO) as “any behavior within an intimate relationship that causes physical, psychological, or sexual harm to those in the relationship,” is experienced by one-third of women globally [1,2]. IPV has consistently been associated with poor mental health, poor sexual and reproductive health, and injury [2]. The United Nations has identified the elimination of IPV against women as a public health priority, and the WHO has called for research to build the evidence base “to address the current lack of information on effectiveness programs for primary prevention” [3,4]. To date, however, there exist few evidence-based primary IPV prevention interventions globally. Further, existing interventions have been largely developed in high-income settings and engage women alone [5-7], men alone [8-11], men and women in independent parallel groups [12-14], men and women together in large groups [15], or communities at large (ie, through mobilization campaigns) [16,17].

In recognition of the gap, we previously developed Ghya Bharari Ekatra (GBE, Marathi for “Take a Flight Together”) [18], the first published couples-based intervention for primary IPV prevention in a resource-limited setting. GBE was designed in Pune, India, to prevent IPV among newly married couples of low socioeconomic status, given the high IPV prevalence in this group and limited availability of support services to them [19]. GBE is culturally sensitive and delivered in the first year of marriage, as opposed to Western countries, where primary IPV prevention traditionally occurs in school years [20,21]. This intervention timing is due to social taboos restricting in-school discussion of intimacy and sexual health in India, the frequent absence of premarital courtship, and the delayed age of individuals’ first sexual relationship, enabling primary prevention to occur later, as well as the recognition that the husband-wife dyad is highly impressionable to behavior modification in early marriage [22]. GBE was delivered by a team of lay peer educators to groups of 3 to 5 couples in 6 weekly 2-hour sessions in the communities in which the couples resided. It makes use of engaging culturally tailored delivery methods (games, role-plays, films, and reflective discussion) to challenge norms and build knowledge and skills in addressing 6 key IPV determinants: limited relationship quality time, poor self-esteem, resilience, communication and conflict management, sexual communication and sexual health knowledge, and conservative IPV norms and definitions.

The development of GBE has been previously described [18]. Briefly, GBE is grounded in the couples interdependence theory [23], which posits that both intrapersonal and interpersonal dyadic processes serve as determinants of a couple’s behavior change, underscoring the need for IPV prevention to engage the couple as a unit. It was developed using a mixed-methods approach to intervention mapping [24], in which intervention components were designed to target determinants of IPV experience and perpetration identified through surveys with newly married women and men, respectively [25,26]. Intervention content and delivery were informed by qualitative research with Indian gender-based violence experts and the lay community [18]. We herein describe the findings of the initial pilot study, which aimed to explore GBE’s acceptance, feasibility, safety, and preliminary efficacy in preventing IPV.

## Methods

### Study Design

This pilot study used a prospective nonrandom design in which groups of 3 to 5 married couples were assigned to receive the intervention or the control condition. Couples assigned to the intervention arm received the 6-session GBE group intervention over a 6-week period plus the ethical standard of care, a list of IPV and mental health support services provided to the female dyadic member. Couples assigned to the control condition received the ethical standard of care alone. Ghya Bharari Ekatra (Marathi for “Take a Flight Together”) is composed of 6 weekly 2-hour sessions, 5 of which are facilitated by a pair of male-female lay community peer educators, with the sixth (focused on sexual communication and sexual and reproductive health) being co-led by medical officers and delivered in gender-concordant groups. Study outcomes assessed included intervention acceptance, feasibility, safety, and preliminary efficacy in preventing IPV, enhancing mental health among women, and reducing alcohol use among men at 3 months.

### Ethics Statement

The study was approved by the National AIDS Research Institute Ethics Committee (Pune, India) and the Emory University Institutional Review Board (Atlanta, Georgia). Written informed consent was obtained from all participants prior to their participation in the study. The trial was registered at ClinicalTrials.gov (registration number NCT03332134) and the Clinical Trials Registry of India (registration number CTRI/2018/01/011596).

### Study Setting and Context

The study was conducted in slum communities in Pune, the second largest metropolis in the western state of Maharashtra, India. According to the most recent census, 22.28% (690,545/3,100,000) of Pune’s populations resides in slums [27,28]. IPV and mental health disorders occur with greater frequency in slums and low-income settings, with national estimates demonstrating IPV prevalence to be 2.5 times higher and morbidity due to mental illness to be 1.3 times higher in the lowest versus highest wealth quintile [19].

### Study Population: Eligibility Criteria and Recruitment

Participants were recruited between January and May of 2018. To be eligible to participate, both members of the couple needed to be 18 years or older; married for ≤1 year; in their first marriage; cohabiting in a slum, chawl, or slum redevelopment community; and fluent in Marathi or Hindi. As GBE was designed to be a primary IPV prevention intervention, couples in which the female member screened positive for physical or sexual IPV using an abridged version of the Indian Family Violence and Control Scale (IFVCS) [29] at baseline were excluded, as were those in the third trimester of pregnancy (since women in the region traditionally return to their natal home during the perinatal period). Both members of the couple had to meet eligibility criteria for the couple to participate.

The participant identification process began with mapping and identifying individual slum communities in Pune (incorporating a geographic buffer around identified communities to prevent contamination). Next, we used a multistaged community sensitization and recruitment process. First, study staff and community-based organizations (CBOs) with whom the Indian Council of Medical Research National AIDS Research Institute (ICMR-NARI) had partnered in prior research studies together contacted community key leaders to notify them of the intent and overall delivery of the intervention. Second, the CBOs and key community leaders then led community sensitization meetings within each slum community to bring community-level awareness of GBE’s aim of fostering healthy relationships and help build intervention support and prevent potential community uprisings that could result from erroneous community speculation of GBE’s intent to preach family planning, publicly disclose household abuse, or treat HIV. Third, CBOs and key leaders identified potential peer educators (to be intervention facilitators) and potentially eligible couple participants. Fourth, study staff then met with families of the couples at their homes to seek initial permission (as per cultural norms). Fifth, study staff met with the couples individually at a private venue of convenience to them (ie, homes, workplaces, community halls) to obtain written informed consent, confirm eligibility, and conduct baseline surveys with each member individually. Once 3 to 5 couples were identified from a particular slum community, the group was assembled and intervention delivery began. In parallel, couples recruited from a neighboring slum community were assigned to the control arm.

### Intervention Preimplementation and Implementation Team

GBE delivery employed a preimplementation phase (led by teams of community leaders and CBOs) and implementation phase (led by peer educators and government medical officers). Community leaders (ie, local government and political figures) and CBOs facilitated community sensitization and entry and helped recruit potential participants and peer educators. Peer educators (one man and one woman per GBE intervention group), who facilitated delivery of the GBE sessions, were lay community people who were married and demonstrated strong oratory, group facilitation, and critical thinking skills and community involvement. They were recruited from various local agencies, including *anganwadis* (government childcare centers), *mitra mandals* (male youth social groups), and CBOs. Session 5, “Sexual Communication and the Sexual Relationship,” was the only session delivered in gender-concordant groups and jointly facilitated by a gender-concordant peer educator and government medical officer. While it may seem counterintuitive that this session was delivered in gender-concordant groups rather than in couples, the strategy was highlighted as imperative by participants involved in the formative GBE design for enhancing acceptance and participant trust [18]. The medical officers were recruited from the government Reproductive Health and Family Planning centers.

### Intervention Facilitation Training: Safeguarding Fidelity

Peer educators underwent a weeklong training by study staff on IPV and gender equality; GBE intent, content, and intervention facilitation methods; methods for establishing rapport; and the safety protocol. The second half of each training day was dedicated to peer educators to practice intervention delivery and receive real-time feedback from the study team. Additionally, during the 6-week intervention delivery period, peer educators received 1-hour weekly paired retraining meetings with a study staff member to practice delivery of the specific module and have module-specific questions answered. These meetings also enabled a space for peer educators to notify study staff of concerns about the safety of particular participants and discuss emotional trauma they themselves were experiencing as intervention facilitators (with study staff facilitating peer referral to support services as necessary). Prior to delivering session 5, “Sexual Communication and the Sexual Relationship,” medical officers received a 2-hour training on the module intent, content, and delivery methods by the research team.

### Intervention

The GBE intervention was delivered to groups of 3 to 5 newly married couples in weekly 2-hour sessions over a 6-week period. It was facilitated by a male-female pair of trained peer educators, conducted in Marathi, and held at a community-based venue (eg, school, community hall, *anganwadi,* CBO) in the slum communities in which the couples resided. The intervention was highly participatory, making use of reflections, discussions, role-plays, games, films, and competitions.

Session 1, “You, Me, and Us: Spending Meaningful Time Together,” aimed to increase the quality time spent together in the relationship and employed a series of self-, couple, and group reflections on the benefits of a marital relationship, the current amount of time the couple spends together, barriers and facilitators to spending quality time with one another, and couple-based planning of strategies to increase quality relationship time.

Session 2, “I’m a Champion: I Can’t Be Broken and I Don’t Accept Defeat,” aimed to increase self-esteem and resilience and employed (1) facilitated group exercises in which participants brainstormed available community and human resources and methods to help address common adversities (eg, job loss, poor health, relationship difficulties) and (2) a guided interview between the two members of a couple in which each gets to know the other on a deeper level (ie, background, hobbies, sources of pride and worry, strengths, modifiable and less modifiable weaknesses), culminating with the recognition of the need to recognize each other as a unit and support the growth of one another.

Session 3, “Building Communication and Conflict Management Skills,” aimed to improve communication and conflict management skills and employed 2 short films that describe the initial hopes, dreams, and expectations of a newly married bride and groom and the subsequent challenges they face and emotions they feel in adjusting to married life in the context of a larger joint, low-income family. This was followed by a powerful peer-led discussion that culminated with the participant group together brainstorming strategies for handling each of the presented situations.

Session 4, “Empowerment of the Couple: Planning Ahead,” aimed to empower both members of the couple through improved goal setting and goal implementation skills and employed (1) couples-based reflections on their personal dreams followed by peer-assisted establishment of and planning for attainable goals and (2) role-plays demonstrating skills to effectively prepare for and participate in a job interview.

Session 5, “Sexual Communication and the Sexual Relationship,” aimed to improve sexual communication and the sexual relationship and was the only module co-led by peer educators and government medical officers and delivered in gender-concordant groups. It employed (1) medical officer–delivered lectures on sexual and reproductive health, (2) a quiz with facilitated discussion that addressed and dispelled commonly held reproductive and sexual health myths, (3) a Snakes and Ladders game adapted to methods for fostering romance in the relationship, and (4) a lecture regarding the importance of and methods for sexual communication with a follow-up question-and-answer period (which made use of an anonymous question box).

Session 6, “A Lens Into Domestic Violence,” aimed to expand participant definitions of behaviors constituting IPV and to challenge subjective norms of IPV occurrence. It employed (1) a facilitated discussion of the different forms and effects of IPV on all members of the family and (2) an exercise in which each participant ranked examples of violence by severity to initiate a discussion about the factors individuals use to define acts of violence, highlighting individual-level differences in conceptualization of violence, and to challenge participants to expand their definitions of IPV and commit to a life of nonviolence. The session ended with a closing video of scrolling photos of the participants engaged in the sessions, presentation to each couple of a framed photo of themselves, announcement of the winner of the competitions embedded in the intervention, and final words from the participants, in which they shared the changes they planned to implement as a result of participating in the intervention.

### Control

Both the control and intervention groups received the ethical standard of care. An appropriate ethical standard of care was designed in consultation with the WHO Ethical and Safety Recommendations for Research on Domestic Violence Against Women [30], and it included the provision of a list of IPV and mental health support services concealed in a phone diary of other services. The IPV support services were included among other services so that if the diary were to be found by the woman’s spouse (a potential IPV perpetrator), he would not be aware of the nature of information provided and her safety would not be jeopardized. The diaries were provided to all female participants at the time of consent regardless of whether they disclosed IPV.

### Outcomes and Data Collection

The main outcomes assessed included intervention acceptability, feasibility, and safety, and secondary outcomes assessed included preliminary efficacy. Outcome evaluation used semistructured group interviews with the intervention participants after each session, semistructured interviews with GBE facilitators after each session, and baseline and 3-month follow-up surveys with individual participants (both control and intervention). The interviews and surveys were conducted by trained research staff. All tools were translated in Marathi prior to use.

Acceptability of the intervention to the dyad was gauged through the postsession semistructured group interviews, wherein the group of participants were collectively asked by the research team about their satisfaction with session content and delivery. The group interview after the sixth (final) session additionally explored satisfaction with the number and duration of the intervention sessions and intervention facilitators, while the follow-up survey included items asking female participants about the perceived adequacy of the study safety procedures. Intervention feasibility was assessed during the postsession interviews with the intervention facilitators, during which they were asked about ease of and challenges with session delivery, their perceptions of participant understanding of the intervention, and logistical problems they encountered with the intervention setting, timing, trainings, and debriefings.

Safety of the intervention was gauged through the follow-up surveys with the women assessing incidents of IPV (using an abridged version of the IFVCS) [29] and an item examining whether the participant felt family or spousal conflict resulted from her participation in the intervention. Additionally, an optional women’s day was held halfway through the intervention period, which female participants could attend to confidentially convey safety concerns to study team members. CBOs and peer educators additionally monitored how the intervention and study were being discussed in the community, a step in the safety protocol designed to defuse concerns early prior to escalation.

Preliminary efficacy was assessed through survey responses measuring changes in (1) past 1-month IPV experience among women (using an abridged version of the IFVCS), (2) mental health among women (using the General Health Questionnaire-12 [GHQ-12]), and (3) past 3-month alcohol consumption among men (using the Alcohol Use Disorders Identification Test [AUDIT]) between baseline and 3-month follow-up.

The process evaluation was conducted by trained study staff members and examined key elements proposed by Saunders et al [31]: fidelity, dose delivered, dose received, participation rate, recruitment, and context. Dose delivered was assessed during each GBE session by the study team member and measured by the number of GBE sessions delivered, percentage of expected content delivered per session, extent to which GBE materials were used in the sessions, and time required to deliver each module. Fidelity was assessed by the extent to which the peer educator and implementation staff training was provided as planned, the extent to which peer educators could deliver each GBE activity during the training session, the extent to which GBE activities were implemented as intended during actual intervention delivery, and exploration of the difficulties that interventionists experienced in delivering GBE activities (through exit interviews at the end of session 6). Recognizing the sensitivity of topics covered, GBE sessions were not recorded to foster open participation. Instead, fidelity was assessed by study staff who were physically present during the sessions (at the back of the intervention venue to minimize disruption) and documented the extent to which each exercise was delivered as intended. Dose received was assessed by a study team member, measured as the level of overall participant engagement (participation) in each module exercise, and through exit interviews with the peer educators and participants at the end of session 6 to assess module-related satisfaction. Participation rate was captured through session attendance tracking and staff contact with participants who missed sessions to assess barriers to participation. Recruitment data were captured through number of participants approached, number who consented, number assessed for eligibility (and reasons for ineligibility where appropriate), and number who completed baseline assessment. Lastly, context was assessed through structured study team observation of the responsiveness of community leaders, community members during sensitization meetings, family members at the time of initial permission, and peer educator and implementation team reporting of the barriers and facilitators encountered in delivering GBE during exit interviews, conducted upon completion of session 6.

### Data Analysis

Intervention acceptance was assessed by exploring trends across the postsession group interviews regarding participant satisfaction with module content and suggested changes. Similarly, feasibility was explored by examining trends across postsession facilitator interviews regarding ease of and difficulty with delivering sessions. Safety was assessed through descriptive analysis of the corresponding items in the follow-up survey. Past 1-month IPV was assessed through the reporting of any physical, sexual, or psychological violence or control using the IFVCS. Mental health was measured using the GHQ-12 total score and past 3-month alcohol consumption was assessed using the AUDIT total score. Intervention efficacy was assessed using the difference in change in each of the 3 parameters from baseline to 3 months between the intervention and control group. Bivariate analyses utilized unpaired *t* tests to test the differences between groups.

## Results

### Participants

**Figure 1:**
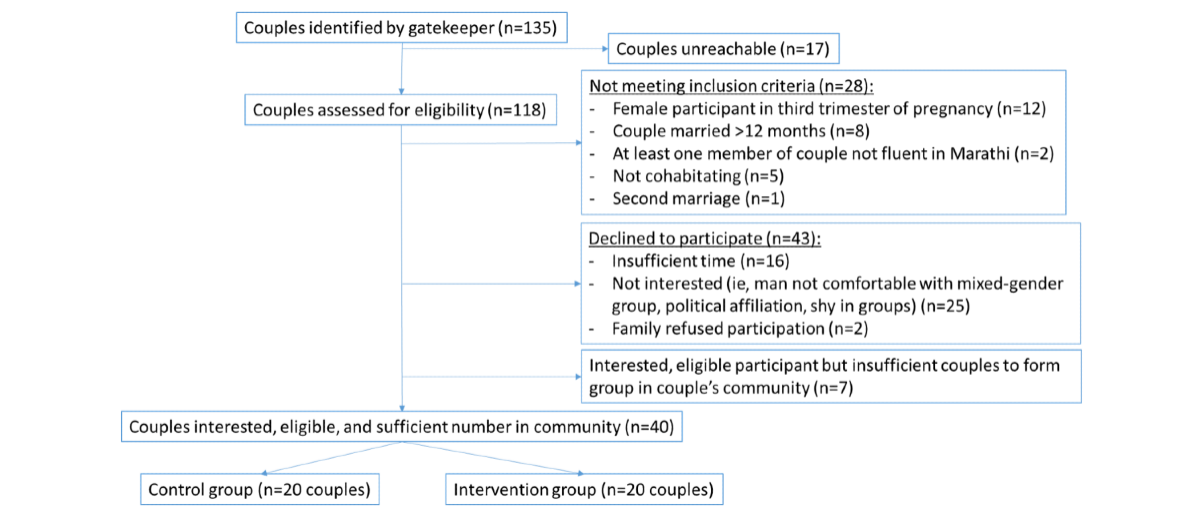
Recruitment and enrollment of participants into Ghya Bharari Ekatra intervention and control groups.

Community gatekeepers identified 135 potentially eligible couples, of which 17 were not reachable, resulting in 118 couples being assessed for eligibility (Figure 1). Of the 118 couples, 28 did not meet eligibility criteria (because the woman was in the third trimester of pregnancy, the couple had been married more than 12 months, a member of the dyad was not fluent in Marathi, the couple was not cohabitating, or it was their second marriage), 43 declined to participate (citing insufficient time, lack of interest, or that their family refused their participation), and 7 were interested and eligible but there were insufficient couples to form an intervention group in the couple’s community. Ultimately, 40 of 83 eligible couples (48%) were enrolled, with 20 assigned to the intervention groups and 20 to the control condition. A total of 5 intervention groups of 3 to 5 couples were formed.

Baseline participant characteristics are described in Table 1. Female participants were an average of 21.6 years (σ=2.8 years) of age, 30% (12/40) were employed, 70% (28/40) had a monthly income of less than Rs 10,000 (US $136.63), and 15% (6/40) were in the first or second trimester of pregnancy. There were statistically significant differences in educational attainment between female participants in the intervention versus control group, with 75% (15/20) of women in the intervention completing secondary or higher education versus 45% (11/20) of women in the control group (*P*=.05). Male participants were an average of 26.4 years (σ=3.1 years) of age, 98% (39/40) were employed, and 60% (24/40) had completed secondary or higher education. There were statistically significant differences in monthly income between male participants in the intervention versus control group, with 85% (17/20) of men in the intervention having a monthly income of more than Rs 10,000 (US $136.63) versus 35% (7/20) of men in the control group (*P*=.001). The majority of couples were Hindu (32/40, 80%), were of a reserved caste (22/40, 55%), lived in joint families (32/40, 80%), and had an average of 5.2 (σ=2.4) members per household. The average marital duration was 6.1 months (σ=3.9 months), with the majority of marriages arranged (29/40, 73%) and occurring within the family (26/40, 65%) and caste (36/40, 90%).

**Table 1:** Baseline participant characteristics.

Characteristic	Women (n=40)			Men (n=40)		
	Intervention (n=20)	Control (n=20)	*P* value	Intervention (n=20)	Control (n=20)	*P* value
Age (years), mean (σ)	21.0 (3.1)	22.2 (2.5)	.16	27.0 (3.3)	25.8 (2.8)	.22
Secondary education or higher, n (%)	15 (75)	11 (45)	.05	14 (70)	10 (50)	.20
Employed, n (%)	5 (25)	7 (35)	.49	20 (100)	19 (95)	—^a^
Women’s income (none), n (%)	15 (75)	13 (65)	.49	N/A^b^	N/A	N/A
Men’s income Rs >10,000^c^, n (%)	N/A	N/A	N/A	17 (85)	7 (35)	.001
Religion (Hindu), n (%)	16 (80)	16 (80)	>.99	15 (75)	16 (80)	.14
Reserved caste, n (%)	10 (50)	12 (60)	.53	—	—	—
Family type (joint), n (%)	14 (70)	18 (90)	.11	—	—	—
Premarital family type (joint), n (%)	6 (30)	9 (45)	.33	—	—	—
Household members, mean (σ)	5.6 (3.0)	4.8 (1.6)	.34	—	—	—
Marital duration (months), mean (σ)	—	—	—	5.5 (3.6)	6.8 (4.2)	.28
Marriage type (arranged), n (%)	14 (70)	15 (75)	.72	—	—	—
Within-caste marriage, n (%)	18 (90)	18 (90)	>.99	—	—	—
Within-family marriage, n (%)	15 (75)	11 (55)	.18	—	—	—
Pregnant, n (%)	2 (10)	4 (20)	.38	—	—	—

^a^Not available.

^b^N/A: not applicable.

^c^Rs 10,000=US $136.63.

### Acceptance

The intervention had high overall acceptance by the participants. At 3-month follow-up, 100% (17/17) of female intervention participants reported they would recommend participating in the intervention to their friends, reporting that the intervention presented them with new information; enabled the couple to build partnership and closeness, understand one another’s goals, and resolve existing misunderstandings; and resulted in their valuing and dedicating more time to their relationship.

Group exit interviews after the individual GBE sessions suggested all 6 sessions were highly accepted, with participants specifically mentioning they enjoyed the participatory nature and integrated games and competitions. Session 3, “Building Communication and Conflict Management Skills,” and Session 5, “Sexual Communication and the Sexual Relationship,” were the most favored. Participants endorsed easily connecting with the session 3 video content, as the dialogs paralleled the frustrations participants faced in their own relationships and family circumstances. Many reported that the videos and subsequent group reflections enabled them to recognize their shared histories and broaden their understanding of their partner’s perspective, thereby fostering cohesion within and among couples in the group. Session 5, “Sexual Communication and the Sexual Relationship,” was highly accepted, as participants reported it filled a key gap in their knowledge and was delivered by trusted health professionals in Marathi. Session 2, “I’m a Champion: I Can’t Be Broken and I Don’t Accept Defeat,” was the least favored, as the delivery of many activities required a higher level of literacy and critical thinking among participants and peer educators. Specific session-specific feedback is presented in Table 2.

**Table 2:** Suggested session modifications to enhance acceptance and feasibility of Ghya Bharari Ekatra intervention delivery.

Session	Suggested modifications
1	The exercise 2 “Prioritizing Time in Relationship” reflections should include additional probes to foster depth of discussion.
2	In the group reflection, peer educators should be reminded to use existing probes about barriers to and strategies for addressing completion of the home assignment, which would enable effective group reflection, even among couples unable to complete the assignment. Exercise 1, “Increased Social Participation,” should directly link participants to community-based organizations hosting social events in place of relying on peers to collect and convey the social event information. The exercise 2 “Enhanced Social Support” group diagramming exercise should be replaced with role-plays and reflections to reduce reliance on literacy and peer critical thinking skills and foster participation. Additionally, scenarios of greater relevance to female group members should be included.
3	To foster depth of discussion and reflection among participants, the film for exercise 2, “Vadal Manache: Film and Discussion,” should incorporate a scene depicting the couple attempting to reconcile differences.
4	Exercise 3 “Getting the Job I Want” mock interviews should be prerecorded to address differences in facilitator skill and comfort level with acting. The resume-building exercise should be replaced with a presentation on the importance of job retention, budget management, and financial resources, as this knowledge is of greater relevance to the intervention population.
5	Two items (No. 7 and No. 10) on the exercise 2 “Misconceptions Quiz” need clarification. Incorporate additional information on HIV, oral sex, and sham infertility treatments.
6	Exercise 1.2, “Expanding My Definition of Violence,” needs additional probes and examples of violence by in-laws and violence related to dowry to foster depth of discussion during reflection. Also, pictures should be added to address literacy challenges with the exercise.

### Safety

No adverse events or safety concerns were raised during the intervention period by participants to research staff. At 3-month follow-up, 100% (17/17) of women reported feeling safe during the 6-week intervention period and none (0/17) reported spousal or family conflicts arising from their participation in the intervention. Study safety procedures were overall well received, with 16 of 17 women reporting the resource diary (which included domestic violence resources) helpful and 15 of 17 women reporting that having the contact information of study staff to be able to contact them at any time was helpful. No women used the women’s day optional session to disclose safety concerns. CBO and peer educator passive monitoring of how the intervention was being discussed in the community also did not identify community tension or safety concerns caused by holding GBE in the communities.

### Feasibility

Feasibility concerns with the delivery of GBE were minor and included (1) challenges with recruitment of male peer facilitators, as many men, while interested in the position, were unwilling to leave their jobs given the temporary nature; (2) challenges with delivering group-based activities (ie, games, group discussions) in sessions where the group size declined from 5 couples to 3 couples due to attrition; (3) delays in initiating session 1 due to the first portion of the study visit being dedicated to obtaining informed consent; (4) occasional temporary difficulties with showing session 3 films due to power outages; and (5) heat exhaustion of facilitators and participants due to lack of fans and air conditioning in most community venues in intense summer temperatures. Sessions 2 and 4 relied on guest key informants (ie, of community-based organizations) who were variably in attendance; while their in-person presence enhanced the quality of the information presented and the discussion, their absence did not significantly impact session delivery, as the material was instead gathered and presented by the peer educators.

### Process Evaluation

GBE was piloted in 5 separate groups of 3 to 5 participants.

#### Dose Delivered

In 4 of 5 groups, all 6 sessions were delivered. One of the 5 groups did not receive session 3 due to participant absence. Expected session content was delivered for all 6 sessions, with the exception of the session 2 group reflection on the home assignment, which was not consistently feasible, as it was dependent on participants having completed the associated home assignment. All intended GBE materials were used across sessions. The average duration of sessions 1 to 6 were 130, 138, 128, 123, 134, and 125 minutes, respectively.

#### Fidelity

The 1-week peer educator and health educator training was provided as planned for all GBE sessions. Overall, peer educators and health educators delivered GBE activities without difficulty during the training. The exception was session 2’s exercises 2 and 3 and the session end summaries, during which facilitators improvised rather than following the script during the training. During the weekly refresher training sessions that preceded each session delivery, the study team focused on retraining the peer educators on the activities they found most challenging. During the actual intervention delivery, all activities were delivered as intended with the exception of session 2’s group reflection (as delivery required that participants had completed the home assignment, which often was not the case), exercise 1 (as it required peers having researched social activities in their communities, which often was not the case), and exercise 2 (as it required a higher level of critical thinking by the facilitator); session 4’s exercise 3 (“The Bad Interview”), as peers had difficulty acting out the bad interview; and session 6’s exercise 1.1, as peers improvised rather than reading directly from the script.

#### Dose Received

Overall participant engagement was high across all session activities. Exceptions were the activities in which the facilitators experienced challenges with delivery (session 1 exercise 2, “Prioritizing the Time Spent in the Relationship”; session 2 exercise 2.1, “Realizing I Have Social Support”; and session 6 exercise 1.2, “Expanding My Definition of Violence”)*.*

#### Participation Rate

A total of 85% (17/20) of couples attended at least 5 of the 6 intervention sessions; 100% (20/20) attended session 1, 80% (16/20) attended session 2, 85% (17/20) attended session 3, 70% (14/20) attended session 4, 85% (17/20) attended session 5, and 85% (17/20) attended session 6. A total of 16% (19/120) of the total sessions were missed due to participant work (7/19, 37%), personal illness (5/19, 26%), family illness (3/19, 16%), the participant being out of town (3/19, 16%), or the participants attending a place of worship (1/19, 5%).

#### Recruitment Data

A total of 118 couples were approached, of which 28 (23.7%) did not meet inclusion criteria, 43 (36.4%) declined to participate, and 7 (5.9%) were not included due to insufficient other eligible couples in their community to form an intervention group. The remaining 40 were assigned to the intervention (n=20) and control group (n=20), with all providing informed consent and completing the baseline assessment (Figure 1).

#### Context

During the preimplementation process, study staff approached key community leaders and law enforcement officials to garner support. Community leaders were highly supportive, helping identify venue space (eg, community childcare centers, community halls) and waiving venue fees, providing electricity, and helping the study team make contacts with peers and potential participants. Law enforcement officials were also highly supportive of the intervention, warned female staff members of security risks at night, and vowed to be alert and responsive if security concerns arose in the community during the intervention. Preimplementation community sensitization meetings had variable attendance (3 to 11 members). Often, only men and mothers-in-law attended, but the meetings enabled study staff to clarify the intent of the intervention, foster dialogue around gatekeeper concerns for the intervention, and conduct an initial eligibility assessment. Preimplementation home visits by study staff to inform the families (particularly the mothers-in-law as household gatekeepers) of the intervention, assess interest, and establish eligibility of the couple were critical to participant recruitment and retention. During the intervention delivery, as the venues were located within often crowded slum communities, ambient noise and maintenance of privacy was often a challenge and required study staff to guard the door and distract onlookers. Additional challenges included adequacy of cooling and emergency lighting.

### Preliminary Efficacy

As the study was a pilot, the sample was small and the follow-up period was short. In this context, there were no reported physical or sexual IPV events in either group but fewer incidents of psychological abuse in GBE participants (3/17, 18%) versus control participants (4/16, 25%) at 3-month follow-up. There was significant improvement in overall mental health of female intervention participants and declines in mental health among the control participants (change in mean GHQ-12 score: –0.13 intervention, 0.13 controls; *P*=.10). Last, among male intervention participants, the mean change in past 3-month alcohol use as measured by the AUDIT was –0.35 in the intervention and –0.11 in the control group (*P*=.74)

## Discussion

GBE is the first documented couples-based primary prevention intervention for IPV developed and piloted in resource-limited settings. The exhaustive pilot evaluation demonstrated high acceptance, feasibility, safety, and preliminary efficacy of GBE in preventing IPV and improving mental health in the female partner. It also identified challenges with participant recruitment and delivery, which could be easily addressed to improve acceptance and fidelity.

The high acceptance, participation, retention rate, safety, and overall feasibility can be attributed to the extensive use of community-based participatory methods during GBE development [18]. Intervention content and delivery was relevant and engaging, as it was designed around stories shared by participants during the development phase and responsive to their request to make the intervention fun and interactive and to have games and competitions. The few changes to enhance acceptance and feasibility (Table 2) would address literacy challenges, further decrease reliance on variance in critical thinking ability by the lay peer educators, and increase depth of reflection and discussions. The high retention was likely also due to the intervention being delivered by lay peer educators recruited from the communities of the participants and acquainted with the participants, the participants knowing one another, the intervention taking place in the communities, and early engagement of key gatekeepers, including the community and families. Early community sensitization, parent engagement, police knowledge, and lack of discussion of relationship IPV also were key to ensuring safety. Noted challenges to feasibility were easily addressable. Difficulties with male peer educator recruitment resulted from the men prioritizing their long-term work over the short-term income generated from attending the 1-week training, 1-hour retrainings, and facilitation of 6 weekly sessions. This barrier would be overcome if the intervention were ultimately implemented and peer facilitators formally hired. To overcome difficulties with recruiting couples of the lowest socioeconomic status (ie, day laborers), compensation should be increased to be commensurate with compensation for lost daily wages. Given the difficulties that arose when group size declined to 3 couples, future delivery should ensure that a minimum of 5 to 6 couples are present per group at the start of the 6-week intervention. Lastly, challenges with electricity shortages and overheating during the summer months could be addressed by selecting venues with backup electric generators and ensuring that fans and video projectors with battery backup are provided.

Although the pilot study used a small sample with a short follow-up, GBE was associated with significantly less reporting of psychological IPV and enhanced mental health among female participants. The prevention of IPV can be attributed to GBE being designed to address the determinants of IPV perpetration and experience identified in these communities [25,26]. There were no reported events of physical or sexual IPV by couples in the experimental or control arm, possibly because these forms of violence are less prevalent in the initial year of marriage [19]. As psychological IPV is often a predecessor of physical and sexual IPV, detection of prevented psychological IPV in the group receiving GBE may be a signal of the longitudinal prevention of physical and sexual forms of abuse in this group as well. The improvement in mental health may have been secondary to the prevention of IPV and strengthening of the relationship quality, as well as other potential social and cognitive mediators modified by the intervention. The absence of a detectable change in alcohol use by the male participants could have been due to low rates of alcohol use during the first year of marriage, the study being inadequately powered to detect these differences between groups, and alcohol harm reduction not being a core focus of GBE*.*

The study had several strengths and limitations. Strengths included the rigor of the process evaluation, the use of a community-based participatory approach that was inclusive of community sensitization predelivery of the intervention, the engaging interactive nature of the intervention, and the community-based delivery by lay peer educators from within the same community. One limitation was the significantly lower enrollment of couples of the lowest socioeconomic status into the intervention versus the control group due to lack of randomization. This bias was likely toward the null, however, as we would expect those of the lowest income to benefit most. A second limitation was the short follow-up period due to the pilot nature of the study, leaving unknown the longitudinal impact of the intervention on participant safety, IPV, mental health, and alcohol use. Future evaluation studies should extend the follow-up period to a minimum of 1 to 2 years to examine sustainability of effect.

In conclusion, GBE has high acceptance, feasibility, and preliminary efficacy in preventing IPV and improving mental health among women. Next steps include refining the intervention content based on the pilot findings and examining intervention efficacy through a large-scale randomized trial with longer follow-up across other regions of India. Implementation data will be collected as part of the trial to inform future dissemination and scaling. If deemed effective, GBE could be scaled across similar settings in Southeast Asia to address the high burden of IPV and mental health disorders.

## Abbreviations

AUDIT: Alcohol Use Disorders Identification Test
CBO: community-based organization
GBE: Ghya Bharari Ekatra
GHQ-12: General Health Questionnaire-12
ICMR-NARI: Indian Council of Medical Research National AIDS Research Institute
IFVCS: Indian Family Violence and Control Scale
IPV: intimate partner violence
